# On the formation of blisters in annealed hydrogenated a-Si layers

**DOI:** 10.1186/1556-276X-8-84

**Published:** 2013-02-15

**Authors:** Miklós Serényi, Cesare Frigeri, Zsolt Szekrényes, Katalin Kamarás, Lucia Nasi, Attila Csik, Nguyen Quoc Khánh

**Affiliations:** 1 Institute of Technical Physics and Materials Science, Research Centre for Natural Sciences, Hungarian Academy of Sciences, P.O. Box 49, Budapest H-1525, Hungary; 2 CNR-IMEM Institute, Parco Area delle Scienze 37/A, Parma 43100, Italy; 3 Institute for Solid State Physics and Optics, Wigner Research Centre for Physics, Hungarian Academy of Sciences, P.O. Box 49, Budapest H-1525, Hungary; 4 Institute of Nuclear Research of the Hungarian Academy of Sciences, P.O. Box 51, Debrecen H-4001, Hungary

**Keywords:** Amorphous Si, Hydrogen, Annealing, IR spectroscopy, Blister

## Abstract

Differently hydrogenated radio frequency-sputtered a-Si layers have been studied by infrared (IR) spectroscopy as a function of the annealing time at 350°C with the aim to get a deeper understanding of the origin of blisters previously observed by us in a-Si/a-Ge multilayers prepared under the same conditions as the ones applied to the present a-Si layers. The H content varied between 10.8 and 17.6 at.% as measured by elastic recoil detection analysis. IR spectroscopy showed that the concentration of the clustered (Si-H)_*n*_ groups and of the (Si-H_2_)_*n*_ (*n* ≥ 1) polymers increased at the expense of the Si-H mono-hydrides with increasing annealing time, suggesting that there is a corresponding increase of the volume of micro-voids whose walls are assumed from literature to be decorated by the clustered mono-hydride groups and polymers. At the same time, an increase in the size of surface blisters was observed. Also, with increasing annealing time, the total concentration of bonded H of any type decreases, indicating that H is partially released from its bonds to Si. It is argued that the H released from the (Si-H)_*n*_ complexes and polymers at the microvoid surfaces form molecular H_2_ inside the voids, whose size increases upon annealing because of the thermal expansion of the H_2_ gas, eventually producing plastic surface deformation in the shape of blisters.

## Background

The electrical and structural properties of hydrogenated amorphous Si, Ge and SiGe are particularly affected by the hydrogen incorporated and its bonding configuration. On one hand, H has proven to be very efficient in reducing the density of open dangling bonds responsible for deep levels in the bandgap. By hydrogenation, their density can be reduced to 10^15^ to 10^16^ cm^−3^ in a-Si [1], which is quite acceptable for device applications, e.g. in photovoltaic solar cells [2]. On the other hand, the H bonding configuration may negatively affect the microstructure of the amorphous lattice. In a-Si, hydrogen is bonded in two modes: as randomly distributed H bonded at isolated network sites (passivating the dangling bonds) and as H bonded in the form of clusters [1,3-6]. Smets found that H is silicon-bonded in hydrogenated di-vacancies [1,7] for low H content. Alternatively, the H clusters are accommodated on the surfaces of voids larger than di-vacancies [4-6]. Nano- and micro-voids have been detected in a-Si [5,7-10] as well as in a-Ge [11]. Such voids are normally present in as-prepared amorphous materials.

As also recently pointed out by Beyer [7], voids are still one of the major defects in hydrogenated a-Si. Being empty spaces, they cause density reduction that can change the refractive index, electronic defect states [7] and anomalous stress distribution especially if filled with H [12] or if they form Si-H platelets [13]. Furthermore, the mentioned H clusters that are situated on the inner surfaces of voids can give rise to potential fluctuations in the bulk that deteriorate the electro-optical properties [14,15]. In a-Si, an increased concentration of Si poly-hydrides, e.g. Si-H_2_ di-hydrides, was seen to increase the optical bandgap [6] and decrease the refractive index [16]. Voids, and related H bonding configurations, are also believed to be involved in the Staebler-Wronsky effect [17,18], i.e. degradation of the hydrogenated a-Si properties upon illumination [1,9].

According to Beyer, cavities in the material are most crucial if they are large enough to accommodate H molecules [7]. In such a case, in fact, hydrogen may desorb as H_2_ with the consequent reconstruction of dangling bonds and Si-Si weak bonds, which causes deterioration of the electronic properties [7]. This work is a contribution in the field of the relationship between H content, H bonding configuration and voids in hydrogenated a-Si single layers deposited by radio frequency (RF) sputtering and subsequently annealed. It was prompted by the need to improve understanding of our previous results about the presence of blisters in hydrogenated a-Si/a-Ge multilayers sputtered in the same way and submitted to annealing with the aim to produce the a-SiGe alloy by Si and Ge diffusion and intermixing [19,20]. It is reported here that annealing of the single a-Si layers causes the voids to grow to such a size to form surface blisters detectable by AFM (atomic force microscopy). By using infrared (IR) spectroscopy, it is shown that the annealing causes the formation of (Si-H)_*n*_ clusters and (Si-H_2_)_*n*_ (*n* ≥ 1) polymers covering the surface of voids. It is then argued that the blisters grow from such voids by accumulation of molecular H_2_ that had formed by reaction between H atoms released from the (Si-H)_*n*_ clusters and (Si-H_2_)_*n*_ (*n* ≥ 1) polymers. The results reported here support and confirm our previous hypothesis that ascribed the blisters in a-Si/a-Ge multilayers to the formation of bubbles containing molecular H_2_[19,20].

## Methods

The a-Si layers have been sputtered at a rate of 6.3 nm/min from a high-purity crystalline silicon target in a high-vacuum sputtering apparatus (Leybold Z400, Fergutec, Valkenswaard, The Netherlands) reaching a base pressure better than 5 × 10^−5^ Pa by a turbo molecular pump. The target was coupled to a RF generator (13.56 MHz) via a network for impedance matching between the generator and its load. The substrate was polished (100) silicon wafer and at a distance of 50 mm away from the target. The layer thickness was approximately 400 nm. Sputtering has been done with a mixture of high-purity argon and hydrogen gases. Both gases have been introduced continuously into the chamber by means of electronically adjustable flow controls. A 1,500-V dc wall potential has been applied to sputter the targets under a plasma pressure of 2 Pa. The samples were annealed in high-purity (99.999%) argon at 350°C for 1 and 4 h.

Controlled layer hydrogenation has been obtained by allowing H to flow continuously into the deposition chamber at different flow rates, namely 0.4, 0.8 and 1.5 ml/min, corresponding to an effective H incorporation in the as-deposited layers of 10.8, 14.7 and 17.6 at.%, respectively, as determined by elastic recoil detection analysis (ERDA). The ERDA measurements were performed with the 1.6 MeV ^4^He^+^ beam at the 5 MeV Van de Graaff accelerator of Budapest on a-Si layers 40-nm thick. The recoiled H signal was collected by an Si detector placed at 10° detecting angle to the beam direction, with the sample tilted 85° to the normal. Almost background-free ERDA spectra for H were obtained by placing a 6-μm-thick Mylar foil in front of the detector to stop the forward-scattered He ions. Further details can be found in [21].

The configuration of the H bonds to Si before and after annealing was evaluated by Fourier transform infrared spectroscopy by employing a Bruker Tensor 37 spectrometer (Bruker, Ettlingen, Germany) with 2 cm^−1^ resolution. All spectra were taken in the 400 to 4,000 cm^−1^ range with a Ge/KBr beam splitter, while the baseline was corrected by an adjusted polynomial function. The index of absorption α(*ω*) is determined from the formula for the *T* transmission coefficient of the film with thickness *d*[22]

(1) $$T\left(\omega \right)=\frac{4T_{0}^{2}e^{-\mathrm{\alpha d}}}{{\left(1+T_{0}\right)}^{2}+{\left(1-T_{0}\right)}^{2}\times e^{-2\mathrm{\alpha d}}}$$

where *T*_0_ is the transmission coefficient of the crystalline silicon substrate. Brodsky et al. verified that the equation is correct within ±10% only for α*d* > 0.1 [22]. *T*_0_ of the single-side-polished substrate was determined experimentally in relation of the transmission through a double specimen to a single one. We found that in the wavenumber region going from 3,000 to 500 cm^−1^, *T*_0_ monotonically decreases from 23% to 16%. This behaviour can be ascribed to the wavelength-dependent light scattering of the rough back side of the wafer.

The concentration *N*_H_ (cm^−3^) of bonded H is obtained by integrating the peaks in the IR spectrum of the absorption coefficient α(*ω*) through the formula [6,22-24]

(2) $$N_{H}=A\int \left[\alpha \left(\omega \right)/\omega \right]\mathrm{d\omega}=A\times \text{I}$$

where *A* (cm^−2^) is a proportionality constant that depends on the vibration mode, *ω* is the oscillatory frequency, or wavenumber (cm^−1^), and *I* is the value of the integral, i.e. the integrated absorption intensity. The integral is extended only to the absorption mode of interest. The total *N*_H_ is calculated either from the wagging mode (at approximately 640 cm^−1^ for Si) or from the stretching mode. In the latter case, since the stretching mode often consists of two peaks at approximately 2,000 and 2,100 cm^−1^, *N*_H_ is given by [23,24]

(3) $$N_{H}=A_{2000}\times I_{2000}+A_{2100}\times I_{2100}$$

Very often, just the integrated intensity *I* is used since it is proportional to the concentration of H bonds to Si apart from a constant value. This procedure is mostly used in this paper. The sample structure was analysed by AFM with a Veeco Dimension 3100 instrument (Veeco Instruments Inc., Plainview, NY, USA) in the tapping mode.

## Results and discussion

Being well established that ERDA provides very reliable absolute values of concentration, the ERDA results about the H concentration have been used to check whether IR can reliably follow the qualitative evolution of the Si-hydrogen bonding configurations as a function of annealing time. To this aim, the relative H concentration, *C*_H_ = *N*_H_/*N*_Si_ with *N*_Si_ the atomic density of Si (5 × 10^22^ cm^−3^), was calculated from deconvoluted IR spectra in the stretching mode range as described in the ‘Methods’ section. Several values for the *A* of the stretching mode to be included in Equations 2 and 3 have appeared in the literature [1,22-25]. It should be mentioned that a local oscillator strength (modified by local field effects and screening by the a-Si matrix) involved in the stretching vibrations leads to different *A* values which are not directly proportional to the hydrogen concentration. The strength of the 2,000 cm^−1^ stretching band saturates with increasing H concentration up to 6 at.%. The 2,100 cm^−1^ vibration continues to increase up to a level of approximately 30 at.%; therefore, at least two different values should be used. Well-accepted values are those of Amato et al. [23] and Langford et al. [24]. They also suggested that instead of two different values, *A*_2000_ and *A*_2100_, an average of them can be used, *A*_av_ = 1.4 × 10^20^ cm^−2^[23,24]. Similar results can be obtained by using the proportionality *A* constant of Brodsky et at. [22] scaled down by a factor of 2 as it was implicitly suggested by them as they wrote that their results are overestimated by a factor of 2 [22,25]. Among the others, Smets et al. suggested instead to use *A*_2000_ = *A*_2100_ = 9.1 × 10^19^ cm^−2^[1].

**Figure 1:**
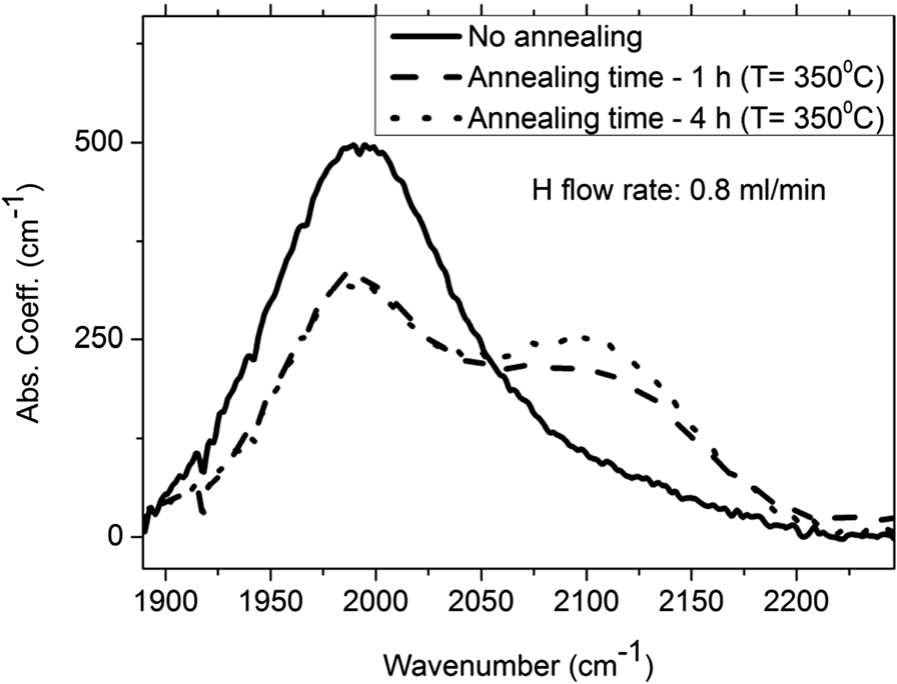
**Typical IR absorption spectra in the SM range for a sample hydrogenated at 0.8 ml/min.** Solid, dash and dot spectra correspond to sample as-deposited, annealed for 1 h and annealed for 4 h, respectively.

**Figure 2:**
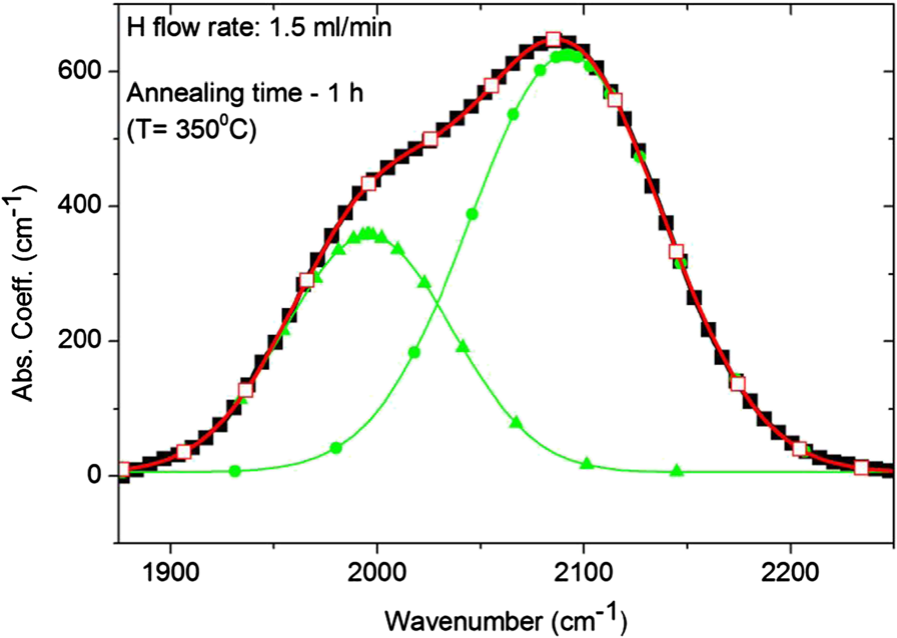
**Results of deconvolution of IR spectra.** Deconvolution of the IR stretching vibration peak into two sub-peaks at 1,996 and 2,092 cm^−1^ in the sample hydrogenated at 1.5 ml/min and annealed at 350°C for 1 h. Similar behaviour is also exhibited by the sample annealed for 4 h. The close square curve is the experimental peak, triangle and dot curves are the two deconvoluted peaks, whereas the open square curve is the fitting to the experimental curve.

Table 1 compares the IR and ERDA results of H concentrations for the case of the a-Si layers hydrogenated with the flow rate of 1.5 ml/min and annealed for different annealing times. The two *A* values mentioned above have been used. The absolute IR concentrations differ from the ERDA ones irrespective of the *A* used. However, the qualitative trend exhibited by the IR and ERDA concentrations is the same, which allowed us to use IR spectroscopy to show the trend of the H bond evolution. Concerning the inexact agreement between the two techniques, it can be due to the lack of a calibration sample having a well-known H content in the ERDA experiments. As a calibration sample, a carbon layer containing H was used. Moreover, the H concentration in the reference sample was determined indirectly from the backscattered spectrum, which may have an uncertainty of 25% [21]. On the other hand, the choice of the *A* plays an important role, as shown by Table 1. In this respect, *A* may also depend on the material type and properties, as discussed in [24]. It should be noticed that the *A* value by Smets yields lower IR concentrations which are more compatible with the measured low absorption coefficient of Figures 1 and 2.

**Table 1:** Comparison between ERDA and IR H concentration in a sample hydrogenated at 1.5 ml/min

**Annealing time (h)**	**H (at.%)**		
	**ERDA**	**IR**	**IR**
		**(A = 1.4 × 10**^**20**^**)[**[23,24]**]**	**(A = 9.1 × 10**^**19**^**)[**[1]**]**
			0	17.5	20.4	13.3
			1	10.9	14.9	9.55
			4	9.9	12.8	8.20

Comparison between ERDA and IR hydrogen concentration in a-Si single layers hydrogenated at 1.5 ml/min as a function of annealing time at 350°C. IR concentrations are calculated with two different *A* values (cm^−2^). See text.

**Figure 3:**
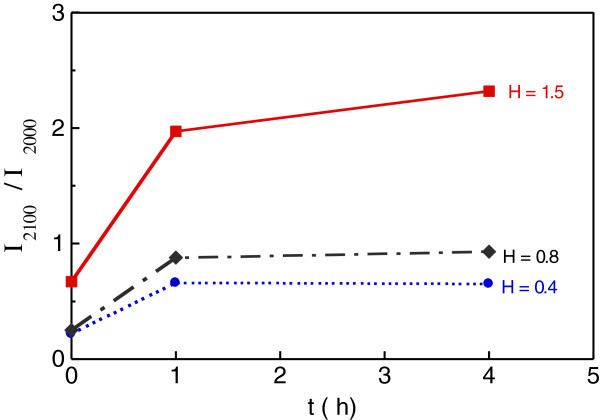
**Plot of *I***_**2100**_**/*I***_**2000 **_**as a function of annealing time for the three values of hydrogenation.** Hydrogenation values: H = 0.4, 0.8 and 1.5 ml/min.

All samples exhibited IR vibration peaks in the wagging, bending and stretching mode ranges. Detailed information about the different H bonding configurations can be extracted from the stretching and bending modes. Figure 1 shows the IR spectra in the stretching mode (SM) range for the as-deposited, annealed for 1 h and annealed for 4 h samples hydrogenated at 0.8 ml/min. It shows a common feature of all samples observed for every applied hydrogenation, i.e. an increase of the contribution of the vibration at higher wavenumber (approximately 2,100 cm^−1^) to the stretching mode with increasing annealing time. Instead, the contribution of the vibration at about 2,000 cm^−1^ decreases. Gaussian deconvolution of the stretching peak of the samples with the highest hydrogen content of 17.6 at.% (H = 1.5 ml/min) and annealed for 1 and 4 h showed that for them the contribution of the vibration at about 2,100 cm^−1^ is even higher than that of the vibration at about 2,000 cm^−1^ (Figure 2). This behaviour is summarised in Figure 3 which gives *I*_2100_/*I*_2000_ as a function of annealing time for the three hydrogenation rates. An increase of the intensity of the stretching peak at high wavenumbers and a decrease of the one at low wavenumbers after annealing have been reported in hydrogenated a-Si obtained by H implantation [8] and by plasma deposition [26]. The increase of the peak at about 2,100 cm^−1^ can be due to the IR activation of H atoms that have occupied interstitial sites, i.e. shallow traps, during sputtering. Because of their low binding energy (0.2 to 0.5 eV) [8], such H atoms may very likely locally rearrange their positions, upon annealing, by breaking weak Si-Si bonds and forming additional Si-H bonds. The latter ones could be of the poly-hydride type, like Si-H_2_, if the rearrangement involves near-neighbouring H atoms. The simultaneous decrease of the peak at about 2,000 cm^−1^, assigned to isolated Si-H mono-hydrides [3-6], would also suggest that previously isolated Si-H bonds may have undergone clustering with formation of (Si-H)_*n*_ groups. As said shortly, they vibrate at approximately 2,100 cm^−1^[4-6,22-24].

According to literature, the vibration mode at approximately 2,000 cm^−1^ is due to the presence of isolated Si-H mono-hydride bonds [3-6,13,16,22-24]. Such mono-hydrides are generally isolated network sites and are associated with H bonded at isolated dangling bonds and vacancies. With increasing H concentration, the hydrogen chemical potential increases, and more complex bonding configurations can form like clustered Si-H groups in the form of Si-H platelets [3,13], (Si-H)_*n*_ groups and poly-hydrides, like Si-H_2_ and chains of them, (Si-H_2_)_*n*_[3-6,16,22-25]. The Si-H platelets should give an IR signature at the frequency of approximately 2,033 cm^−1^[3]. An IR absorption peak that could be ascribed to Si-H platelets was only observed in the as-deposited sample hydrogenated at the lowest rate of 0.4 ml/min that exhibited a peak at 2,054 cm^−1^. The poly-hydride bonds instead IR vibrate at approximately 2,100 cm^−1^[4-6,22-24]. The clustered (Si-H)_*n*_ groups also vibrate at approximately 2,100 cm^−1^[4-6,13,16,22-24]. The Si-H mono-hydrides do not yield any bending mode vibration, whereas Si-H_2_ and chains of it, (Si-H_2_)_*n*_, do [4-6,13,16,22-24]. This was used to check the contribution of the latter poly-hydrides to the stretching mode absorption at approximately 2,100 cm^−1^.

**Figure 4:**
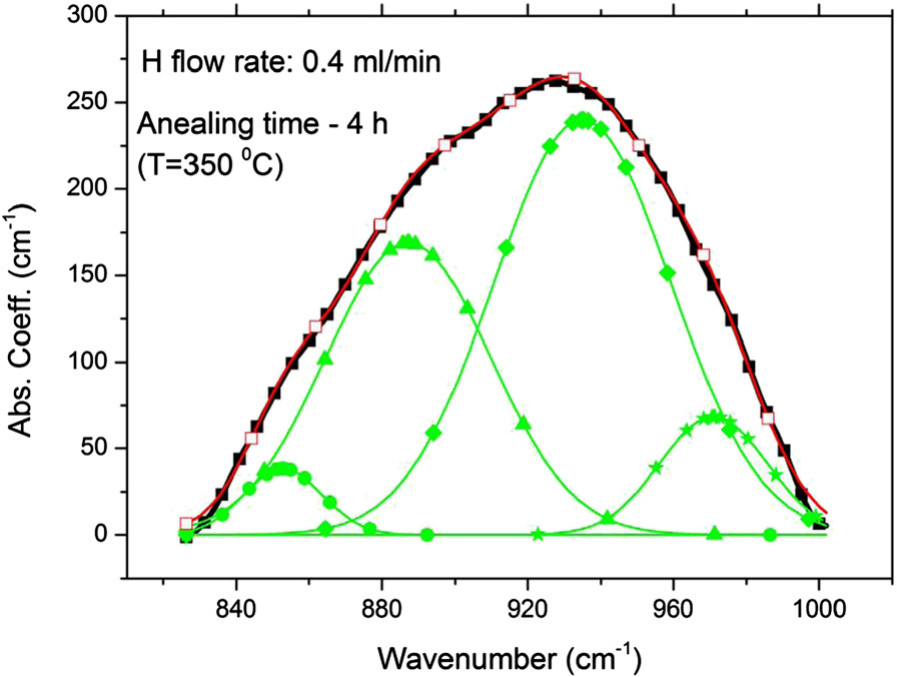
**IR bending mode range.** Gaussian deconvolution of a broad IR peak between approximately 835 and 1,000 cm^−1^ for the case of the sample hydrogenated at a rate of 0.4 ml/min (H content = 10.8 at.%) and annealed for 4 h. The two peaks at 853 (circles) and 887 (triangles) are due to the bending mode oscillations of Si di-hydrides. See text.

The bending mode absorption peak was observed in all samples although included in a broad peak. An example of deconvolution of one such broad peak is shown in Figure 4 for the case of the sample hydrogenated at a rate of 0.4 ml/min and annealed for 4 h. The broad peak is fitted by four Gaussians peaked at 853, 887, 936 and 971 cm^−1^. The former two peaks are the bending mode vibrations of the Si-H_2_ di-hydrides, i.e. Si-H_2_ and (Si-H_2_)_*n*_[4]. The other two peaks at the higher wavenumbers of 936 and 971 cm^−1^ have to be ascribed to Si-O vibrations [4]. The bending vibrations at 887 and 853 are usually assigned to Si-H_2_ di-hydrides and to chains of it, (Si-H_2_)_*n*_, respectively [4,5,16,22-26]. Their presence in the annealed layers is thus confirmed by Figure 4. However, the fitting of Figure 4 shows that the concentration of the (Si-H_2_)_*n*_ chains is some percentage (9.2% in Figure 4) of that of the single Si-H_2_ di-hydrides. It can thus be concluded that besides the mono-hydride clusters (Si-H)_*n*_, the Si-H_2_ di-hydrides, as well as the (Si-H_2_)_*n*_ chains (though in a reduced percentage), contribute to the stretching absorption at about 2,100 cm^−1^. All such Si-hydrogen complexes are reported to reside on the surfaces of voids [4-6,8-16,22-26].

**Figure 5:**
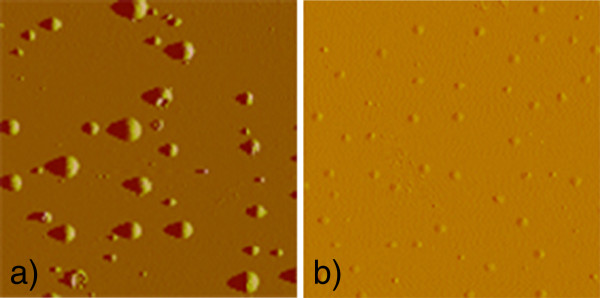
**AFM images of surface blisters.** (**a**) Sample hydrogenated at 1.5 ml/min and annealed for 1 h (scan size 40 μm) and (**b**) sample hydrogenated at 0.4 ml/min and annealed for 4 h (scan size 10 μm).

Figure 3 shows that in the as-deposited samples, H is bonded to Si mainly as mono-hydride Si-H, very likely saturating dangling bonds or occupying di-vacancies, as said earlier. Since *I*_2100_/*I*_2000_ is not zero (Figure 3), a certain amount of H also forms the mentioned complexes residing on the surfaces of nano-voids expected to be present in the amorphous host Si material. Nano-voids, with a size of a few nanometers, have been detected in a-Si irrespective of the growth method [5,8-10]. The significant increase of the concentration of the clustered (Si-H)_*n*_ groups and poly-hydrides already after 1-h annealing suggests that the size of the nano-voids has increased, thus offering a larger surface for formation of those complexes. This phenomenon is greater in the samples with the highest H content (1.5 ml/min) for which *I*_2100_/*I*_2000_ > 1 for annealing times ≥1 h (Figure 3). The size increase of the nano-voids may have occurred by an Ostwald ripening mechanism [8,27] whereby small cavities coalesce forming larger ones. Parallel to the increase of the density of the mentioned H complexes in the annealed samples also is the presence of surface blisters, examples of which are shown in the AFM images of Figure 5. The height, size and density of the blisters increase with increasing annealing time and/or H content, similar to what was already observed in a-Si/a-Ge multilayers [19,20], i.e. they show the same behaviour as a function of the annealing temperature as the concentration of the H complexes does. It should be noticed that the height of the blisters remains below 100 nm, and therefore, they do not increase the nonspecular scattering of the micrometre waves in the stretching mode regime in the IR experiments.

Table 2 reports the total integrated intensity of the stretching mode, *I*_SM_ = ∫ *α*(*ω*)/*ωdω* obtained by summing up the integrated intensities of the two deconvoluted peaks at approximately 2,000 and 2,100 cm^−1^, as a function of annealing time for the three rates of hydrogenation. It shows that the total amount of Si-hydrogen bonds of any type, i.e. the total amount of bonded H, decreases by increasing the annealing time, which suggests that the annealing caused the break of some of the bonds of H to Si. H release from the isolated mono-hydrides is expected to be less likely as they represent the deepest binding sites [13]. If release occurred, H atoms would occupy interstitial positions wherefrom they might diffuse toward the voids and ensure H supply in the environment of blisters. The clustered Si-H groups and polymers decorating the walls of the voids have instead a smaller binding energy [13] and are expected to easily liberate their H into the voids themselves where H atoms may react to form molecular H_2_. According to [26,28], H evolution, i.e., break of Si-hydrogen bonds, already starts at temperatures of 250°C [26] or 150°C [28], which are much lower than the annealing temperature used here. The molecular H_2_ in the gas state inside the nanocavities expands upon annealing with consequent increase of the volume of the nanocavities, which would favour their coalescence, leading to bigger and bigger voids. Such bigger voids offer larger inner surfaces for the formation of additional clustered mono-hydrides and (Si-H_2_)_*n*_, *n* ≥ 1, polymers which will further contribute to the release of additional H to be transformed into H_2_. Eventually, the voids will reach such a big size to cause a lift-off of the layers with the formation of surface blisters, as observed by AFM. The blisters correspond therefore to bubbles containing molecular H_2_. They have developed from microscopic cavities, decorated by clustered mono-hydrides and (Si-H_2_)_*n*_, *n* ≥ 1, complexes, which have increased their volume because of the increase of the inside pressure due to the thermal expansion of the H_2_ gas upon annealing. It was seen in previous works on a-Si, a-Ge layers and a-Si/a-Ge multilayers that for annealing time and/or temperature higher than those considered here, further degradation of the layer surface occurs by explosion of the blisters [19,20].

**Table 2:** **Total integrated intensity (cm**^**−1**^**) of the IR stretching mode**

**Annealing time (h)**	***I***_**SM**_**(cm**^**−1**^**)**		
	**H = 0.4 ml/min**	**H = 0.8 ml/min**	**H = 1.5 ml/min**
0	12.8	30.8	72.1
1	11.4	26.8	52.5
4	10.5	24.2	45.1

Total integrated intensity (cm^−1^) of the IR stretching mode, *I*_SM_, as a function of annealing time for the different hydrogenation rates.

## Conclusions

The origin of surface blisters that form in hydrogenated RF-sputtered a-Si layers submitted to annealing has been investigated by studying the evolution of the Si-hydrogen bonds by means of IR spectroscopy. By increasing the annealing time and/or H content, the blister size increased. Correspondingly, IR spectroscopy showed that the density of the isolated Si-H mono-hydrides decreased, while the concentration of the clustered (Si-H)_*n*_ groups and (Si-H_2_)_*n*_, *n* ≥ 1, polymers increased. As both these complexes reside on the inner surfaces of voids, it is concluded that their accumulation at such surfaces favours the void size increase. It was also seen that the total amount of bonded H decreased upon annealing, suggesting that some H is released from its bonds to Si. The H liberated from the (Si-H)_*n*_ groups and (Si-H_2_)_*n*_ polymers decorating the void surfaces is expected to form molecular H_2_ within the voids. The expansion of the H_2_ gas would cause further growth of the voids up to a size able to produce surface blistering.

## Competing interests

The authors declare that they have no competing interests.

## Authors’ contributions

MS grew the samples by sputtering, suggested and coordinated the experiment. CF coordinated the interpretation of the results and drafted the manuscript, ZS carried out the IR measurements. KK participated in the IR data elaboration. LN made the AFM work. AC carried out the sample heating experiments. NQK performed the ERDA measurements. All authors read and approved the final manuscript.

## Authors’ information

MS is a scientific adviser at the Institute of Technical Physics and Materials Science, Research Centre for Natural Sciences, Hungarian Academy of Sciences, Budapest, Hungary. CF is a senior scientist at the IMEM Institute of the Consiglio Nazionale delle Ricerche, Parma, Italy. ZS is a PhD student and young researcher at the Institute for Solid State Physics and Optics, Wigner Research Centre for Physics, Hungarian Academy of Sciences, Budapest, Hungary. KK is a research professor at the Institute for Solid State Physics and Optics, Wigner Research Centre for Physics, Hungarian Academy of Sciences, Budapest, Hungary. LN is a researcher at the IMEM Institute of the Consiglio Nazionale delle Ricerche, Parma, Italy. AC is a senior research associate at the Institute of Nuclear Research of the Hungarian Academy of Sciences, Hungary. NQK is senior scientist at the Institute of Technical Physics and Materials Science, Research Centre for Natural Sciences, Hungarian Academy of Sciences, Budapest, Hungary.
